# Effect of Baduanjin exercise intervention on cognitive function and quality of life in women with breast cancer receiving chemotherapy: study protocol of a randomized controlled trial

**DOI:** 10.1186/s13063-021-05355-w

**Published:** 2021-06-19

**Authors:** Xiao-Lin Wei, Ru-Zhen Yuan, Yong-Mei Jin, Shu Li, Ming-Yue Wang, Jie-Ting Jiang, Cai-Qin Wu, Kun-Peng Li

**Affiliations:** 1 School of Nursing, Shanghai University of Traditional Chinese Medicine, 1200 Cailun Road, Shanghai, 201203 China; 2 Nursing Department, The Seventh People’s Hospital Affiliated to Shanghai University of Traditional Chinese Medicine, 358 Datong Road, Shanghai, 200137 China; 3 Department of Respiratory Medicine, The Seventh People’s Hospital Affiliated to Shanghai University of Traditional Chinese Medicine, 358 Datong Road, Shanghai, 200137 China; 4 Department of Neurorehabilitation, The Second Rehabilitation Hospital of Shanghai, 860 Changjiang Road, Shanghai, 200441 China

**Keywords:** Baduanjin exercise, Breast cancer, Cognitive function, Quality of life

## Abstract

**Background:**

More than 50% cognitive impairment was reported by cancer patients before and after medical treatment. However, there are no effective interventions to manage the cognitive problem in women with breast cancer. This pilot study was designed to evaluate the protective effect of Baduanjin exercise on cognitive function and cancer-related symptoms in women with early-stage breast cancer undergoing chemotherapy.

**Method:**

A single-blinded, randomized control trial was designed. The trial will recruit 70 patients with early-stage breast cancer scheduled to receive chemotherapy from Shanghai in China. All participants will be randomly assigned to (1:1) the supervised Baduanjin group (5 times/week, 30 min each time) or the wait-list control group for 3 months. The effect of Baduanjin exercise intervention will be evaluated by outcome measures including subjective and objective cognitive function, symptoms (fatigue, depression, and anxiety), and health-related quality of life at pre-intervention (T0), 8 weeks (T1), and 12 weeks (T2). The PCI score in the FACT-Cog as the primary cognitive outcome will be reported descriptively, while effect sizes and 95% confidence intervals (CIs) will be calculated. The collected data will be analyzed by using an intention-to-treat principle and linear mixed-effects modeling.

**Discussion:**

This is the first randomized clinical trial to investigate whether Baduanjin exercise will have a positive role in improving cognitive function in women with breast cancer receiving chemotherapy. If possible, Baduanjin exercise will be a potential non-pharmacological intervention to manage cognitive dysfunction and promote survivorship care among breast cancer survivors.

**Trial registration:**

Chinese Clinical Trial Registry (ChiCTR) ChiCTR2000033152. Registered on 22 May 2020

**Supplementary Information:**

The online version contains supplementary material available at 10.1186/s13063-021-05355-w.

## Background

Cognitive dysfunction is a frequently reported side effect that cancer patients experience resulting from cancer and medical treatment (e.g., surgical resection, chemotherapy, local radiation therapy, and endocrine therapy), showing a cognitive decline in the field of memory, processing speed, attention, and executive functions [1–4], and it is also known as cancer-related cognitive impairment (CRCI). More than 50% of breast cancer patients have subjective cognitive complaints, and 15–25% of cancer patients have objective cognitive dysfunction [5, 6]. Cognitive impairment may persist for years beyond the completion of treatment for some patients [7]. Although the cognitive change is generally mild to moderate, it has a negative impact on the physiological condition, activities of daily living, professional achievement [8], treatment adherence [9], and overall quality of life of cancer survivors in the context of long-term cancer care [10, 11].

Currently, the biological mechanisms of CRCI are not identified, but there are several results based on animal and clinical studies. The mechanisms include (1) increase in the levels of chemotherapy-induced pro-inflammatory cytokines (e.g., IL-6, TNF-α) [12, 13]; (2) effects of chemotherapy-induced neurotoxicity on the brain structure and function (e.g., white matter damage [14], inhibition of neurogenesis [15], and changes in neurotransmitter [16]); (3) changes in the blood vessels and blood flow in the central nervous system [17]; and (4) direct and oxidative DNA damage [18]. In addition, pervasive fatigue and negative emotions may contribute to increased susceptibility of cognitive impairment [19]. It is unclear whether the cognitive impairment results from one mechanism or a combination of multiple effects.

However, there are currently no effective interventions to adequately alleviate or reduce the risk of cognitive impairment. Various forms of physical activity have been studied to improve cognitive function in healthy older adults and people with mild cognitive impairment (MCI) [20, 21]. Emerging preliminary results have demonstrated that physical activities are a potential intervention to improve cognitive function, but conflicting results have been reported. Two pilot studies [22, 23] showed that aerobic exercise improved objective cognition in patients with breast cancer after primary treatment. Hartman et al. [22] found that a 12-week exercise intervention improved the processing speed in patients diagnosed with breast cancer in the preceding 2 years, but there was no significant improvement in self-reported cognitive function. Another study showed that a 24-week supervised and home-based aerobic exercise program demonstrated similar results in postmenopausal women with early-stage breast cancer [23]. Nevertheless, Galvão et al. [24] showed that aerobic exercise plus resistance training improved subjective cognitive function from a subscale of health-related quality of life scale (EORTC-QLQ-C30) and decreased the level of C-reactive protein (CRP). The conflicting results of previous studies are likely due to the insufficient sample sizes and heterogeneity of types and timing of exercise interventions. Moreover, most of the studies used subjective cognitive scales instead of multidimensional measures of objective cognitive function. More rigorous and replicable clinical trials are warranted to prove potential benefits of physical exercise in breast cancer patients with cognitive problems.

In addition to aerobic exercise, research based on mind-body therapy have also achieved preliminary results in improving cognitive function among cancer patients [25–27]. In breast cancer survivors, mindfulness-based exercise may be superior to gentle exercise or survivorship support in terms of improving self-reported cognitive function and distress [25]. Oh et al. [26] demonstrated that a 10-week medical Qigong program significantly improved subjective cognition with medium effect size. Baduanjin, a traditional Chinese fitness exercise combining meditation and repetitive movement with low intensity [28, 29], is considered to be an effective exercise in promoting health [28]. Recently, a review [30] supported that Baduanjin exercise may be effective as an adjunctive rehabilitation method for improving cognitive function, but the effect of Baduanjin exercise on CRCI needs to be further explored.

Therefore, the primary purpose of this article is to describe the protocol of a randomized control trial to examine whether Baduanjin exercise program is a protective measure in improving cognitive function in chemotherapy-exposed patients with early-stage breast cancer.

The main aims of the trial are as follows:

1. Whether a 3-month Baduanjin intervention improves subjective and objective cognitive function of breast cancer patients during chemotherapy
2. Whether a 3-month exercise intervention improves quality of life and cancer-related symptoms such as fatigue, depression, and anxiety
3. Whether there is an association between subjective or objective cognitive function, symptoms, and quality of life

## Methods/design

### Study design

**Fig. 1:**
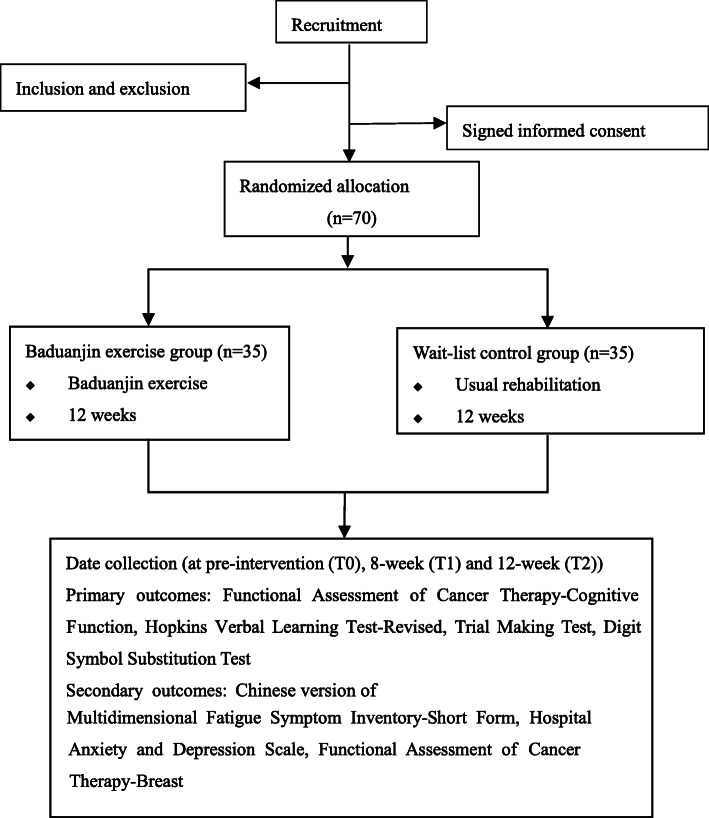
Overview of the study

This is a single-blinded randomized control trial (RCT) with two study arms, including a supervised Baduanjin intervention group and a wait-list control group. An overview of the study is displayed in Fig. 1. The overall development of the study protocol followed the Standard Protocol Items: Recommendations for Interventional Trials (SPIRIT) guidelines [31]. The protection of human subject was approved by the Ethics Committee of the Seventh People’s Hospital of Shanghai University of Traditional Chinese Medicine.

### Study setting

Participants are recruited from the three affiliated hospitals of Shanghai University of Traditional Chinese Medicine, including Seventh People’s Hospital, Longhua Hospital, and Yueyang Hospital of Integrated Traditional Chinese and Western Medicine. The outcome measures are evaluated in a quiet room in the hospital to avoid interference.

### Recruitment

Recruitment of breast cancer patients started in 1 June 2020. Researchers can screen breast cancer patients who meet the inclusion and exclusion criteria through electronic medical records, and provide information brochure of the study, as well as possible benefits of the exercise and the relevant safety during the trial, in order to obtain informed consent. In addition, recruitment posters will be posted in the hospitals and via social media such as WeChat, an online communication app. After consent is obtained, the research team will screen the candidates with patients’ questionnaire and neuropsychological testing. Other parameters, including height and weight to calculate body mass index (BMI), will also be recorded.

### Inclusion criteria

The inclusion criteria meeting this study are as follows:

1. Female patients newly diagnosed with stage I to III breast cancer
2. Scheduled to receive adjuvant chemotherapy
3. Aged 40 to 75 years
4. Using WeChat
5. Sufficient fluency of the Chinese language
6. Willing to participate in the study and be randomly assigned

### Exclusion criteria

The patient will be excluded for meeting any of the following criteria:

1. Disease recurrence or metastasis
2. Known conditions and/or diseases that impact cognition (e.g., Alzheimer’s disease, dementia, or other psychological diagnoses)
3. Disorders that might preclude exercise participation (e.g., fracture, arthritis)
4. Current/planned participation in mindfulness-based exercise programs (e.g., TaiChi or Qigong)

### Randomization and blinding

Participants will be randomly assigned to 1 of 2 groups—an intervention group or a control group—in a 1:1 ratio using the SPSS 24.0 software. The allocation of the result will be put into sealed, opaque envelopes by a study administrator not involved in the study, and the group numbers will be subsequently disclosed. Given that Baduanjin exercise is a well-known exercise to the study population, blind method is conducted to assessors only, not physicians or patients. In addition, all participants will be asked not to discuss the practice with each other to minimize bias. The assessor will also be instructed not to acquire the participant’s exercise information. Participants are allowed to withdraw without giving a reason at any time. If any patient experiences adverse effect during exercise, such as palpitation or severe upper limb lymphedema, but still wishes to participate, there will be a therapist available who will evaluate whether the patient can continue on the study.

### Interventions

Upon completion of baseline testing and randomization, participants are notified of the group assignment by the unblinded project director. At that time, a participant’s group assignment and baseline assessment will be conducted within 3 days of beginning chemotherapy.

### Baduanjin exercise group

Baduanjin exercise is conducted according to the standards promulgated by the General Administration of Sport in China in 2003, which consists of 10 postures (including the beginning and ending posture). A professionally trained Qigong specialist will uniformly train subject researchers to learn Baduanjin. Researchers will then conduct on-site instruction to guide patients and correct their movements. Participants will also be provided with video demonstration. Before the first cycle of chemotherapy, the patients will conduct the first session in the hospital, then follow the video workout at home. Recommended training time is 5 times a week, for half an hour each time, during the 12-week study period. The program begins with stretching the joints, inhalation and exhalation for 2 min respectively, and two 12-min Baduanjin sessions, followed by 2 min of muscle relaxation exercises. Patients’ log will be recorded including any obstacles during exercise at home. When the patients go to the hospital to receive chemotherapy, the feedback of exercise log will be collected and checked. To promote participant retention, we will follow up and encourage the patients through WeChat twice weekly, asking the factors that hinder their activities and solving it in time. In addition, small gifts will be given to patients (e.g., towels and toothbrushes) to promote their exercise enthusiasm at each assessment.

### Wait-list control group

Patients randomized to the control group are given a face-to-face health education according to the handbook to maintain their usual healthy lifestyle. Disease-related questions raised by the patients will be directly communicated or answered through WeChat online. Meanwhile, researchers need to ask patients about their conditions twice a week. Patients can exercise the affected limb through exercises including abduction, forward flexion, backward extension, internal rotation, and wall climbing, according to the guideline and standard for the diagnosis and treatment of breast cancer set forth by the Chinese Anti-Cancer Association (2019 edition), and the patients are not asked to conduct other aerobic activities. The supervised Baduanjin exercise for participants will be offered if they wish after the completion of study.

### Study assessments

**Fig. 2:**
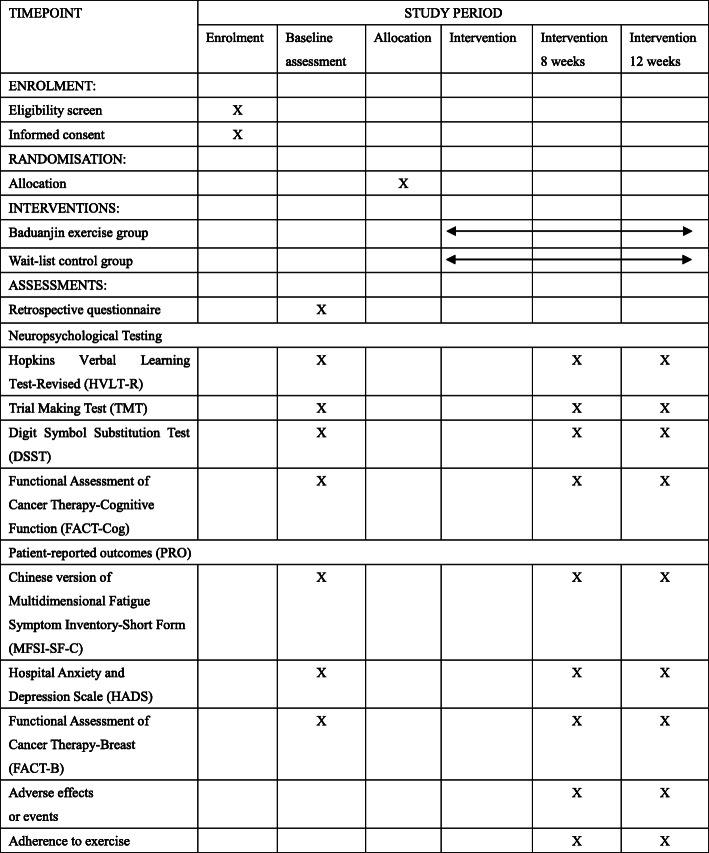
Standard Protocol Items: Recommendations for Interventional Trials (SPIRIT) diagram of enrolment, treatment, and assessments over intervention time

Cognitive ability and several patient-reported outcome (PRO) questionnaires will be collected as primary outcome and secondary outcomes, respectively. These outcome assessments will be carried out by professional researchers at pre-intervention (T0), 8 weeks (T1), and 12 weeks (T2) during intervention. The most important primary outcome is cognitive ability, which is assessed using Cancer Therapy-Cognitive Function (FACT-Cog). If there is a 7-point improvement in the FACT-Cog PCI score between the groups, then the intervention will be thought to have tended to have a beneficial effect based on minimal clinically important difference (MCID) [32]. Demographic information collected via interview pre-intervention include patients’ age, level of education, and economic status. Disease information is retrieved from medical records by two trained study coordinators (M-YW, J-TJ). The schedule of study outcome assessments is outlined in Fig. 2.

### Cognitive ability

Patients’ subjective feelings are the most relevant measures of clinical significance, and cognitive complaint reported by patients is the main outcome indicator. The Functional Assessment of Cancer Therapy-Cognitive Function (FACT-Cog) was developed by Wagner et al. [33] to assess subjective cognitive function, and revised Chinese version will be used in this study [34]. The scale was divided into 4 dimensions including 37 items: perceived cognitive impairment (PCI), perceived cognitive ability (PCA), evaluation of others, and impact on quality of life (QoL). The higher the score of the overall cognitive impairment, the better the cognitive function. Cronbach’s α coefficient is from 0.87 to 0.96 with good internal consistency.

In addition, the International Cognition and Cancer Task Force (ICCTF) [1] proposed and considered the objective neuropsychological test battery to be the gold standard for evaluating cognitive function of CRCI. The Hopkins Verbal Learning Test-Revised (HVLT-R) [35] is a test used to assess verbal learning and memory. The Chinese version of HVLT consists of a 12-word list, and the researcher will read them aloud. The patient needs to recall as many words as possible for three consecutive learning trials (total recall). After 20 min, the patient will be asked again to recall (delayed recall). Lastly, a list of 24 words will be read by the researcher, and the patient will be required to declare and answer whether the particular word is mentioned in previous list (recognition). At the subsequent follow-up, a parallel version of HVLT (the 12-word list with different words) is applied to replace the original version. In addition, executive function and attention are evaluated by the Trail-Making Test (TMT) [36] and Digit Symbol Substitution Test (DSST) [37], respectively (see Table 1).

**Table 1:**
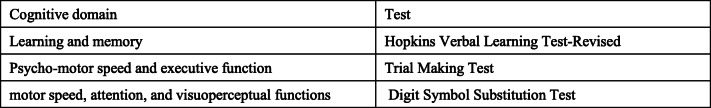
Comprehensive neuropsychological test

### Patient-reported outcomes

Patients will complete several patient-reported outcome questionnaires aimed to evaluate subjective fatigue (Chinese version of the Multidimensional Fatigue Symptom Inventory-Short Form (MFSI-SF-C) [38]), mood (Hospital Anxiety and Depression Scale (HADS) [39]), and health-related quality of life (Functional Assessment of Cancer Therapy-Breast (FACT-B) [40]) (see Table 2).

**Table 2:**
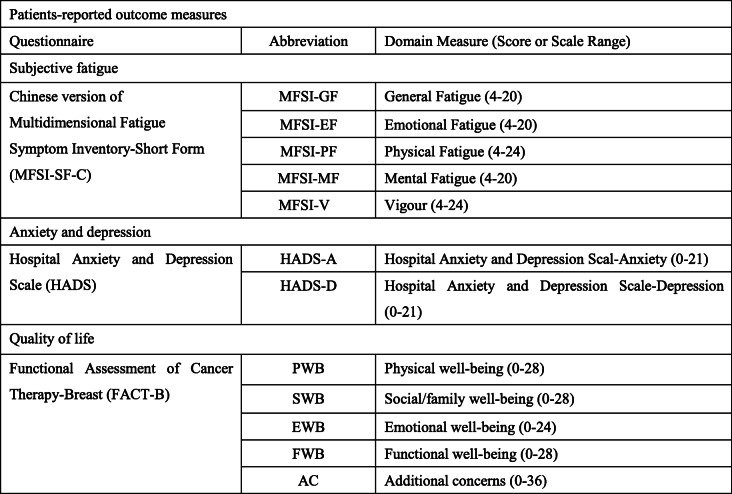
Patient-reported outcome measures at baseline and follow-up

### Safety

Adverse events occurred during the study should be truthfully recorded in the adverse event record form, including symptoms, occurrence time, duration, and treatment measures. Adverse events were divided into general adverse events, major adverse events, and serious adverse events according to their severity. A report form should be completed for a serious adverse event (SAE). Evaluation parameters include: level 1 (safe and no adverse effects), level 2 (relatively safe, mild adverse effects, and continued training with no need to do any treatment), level 3 (insecure, moderate adverse effects, and continued training after treatment), and level 4 (suspended study due to serious adverse effects). An adverse event that meets the criteria for SAE will be reported to the local Institutional Review Board.

### Trial monitoring

The trial is considered low risk without stopping rules, so Investigational Medicinal Products will not be used. The independent Trial Oversight Committee (TOC) will review the protocol and the statistical analysis plan, and review recruitment of patients, compliance with protocol, adverse events or effects, and the collection of complete data. The research staff are independent experts specializing in clinical trials, cancer nursing, or statistics. The Trial Management Committee will supervise the trial procedures and progress, and the outcomes will be reported regularly.

### Protocol amendments

The sponsor is responsible for submitting materials to the local ethics committee. During this process, the principal investigator may be trained in any program modification. If necessary, any retraining shall be provided.

### Sample size

The sample size was estimated in this study by using perceptive cognitive impairment as the main effect indicator, which refers to the results of Oh et al. [26]. Using the G*Power 3.1 software, 58 cases of the total sample can be calculated with a test power of 0.80. Considering the 20% dropout rate, the total sample size for this study is about 70 patients randomized to the intervention group or the control group.

### Data collection

All the data will be collected from participants, including those who discontinue the intervention. Most of the outcome data will be collected via the provided paper-and-pencil measures (anonymised by participant ID). If participants are interested in electronic measures (anonymised by participant ID), these will be provided.

The cognitive assessors will conduct a comprehensive training in scoring the measures. Interrater reliability will be determined, and discrepancies will be resolved by consensus. After participants have been randomized, dropout and premature termination from the two groups at any point will be recorded along with relevant reason. Participants can choose to withdraw and will not be affected at any time while declining to participate or withdrawing from the trial. In some cases, participants will discontinue the intervention, such as illness, progression of their disease, or inability to engage despite adjustment of the program. Participants who are withdrawn from the intervention during the trial will not continue to follow-up but will not be replaced.

### Data management

When the data collection is completed, all paper data will be converted into electronic data according to the case report form (CRF). All data will be independently recorded by two researchers (M-YW, J-TJ) using the EpiData software version 3.1. The software automatically checks inconsistent or questionable data according to the check results, and generates the data question form (DQF), which is handed over to the investigator for review. After all the data are confirmed, checked, and stored in an electronic database, the identifiable information (e.g., real name) of the participants will not appear in the relevant reports of the trial to protect their privacy. Only researchers directly involved in the analysis of the RCT will have access to the final trial data set, which will only contain coded data.

### Data analysis

Demographics and other characteristics will be reported descriptively to summarize the distribution of all variables via the statistics software (SPSS 24.0). The means and SD will be calculated for continuous normally distributed variables and medians and ranges for non-normally distributed variables. Categorical variables will be presented by absolute numbers and percentages. All cases for both baseline and follow-up measurements will be analyzed for the different outcomes. If missing values exceed 10%, we will impute missing values on covariates by using multiple imputation.

According to the intention-to-treat principle, we will compare the number of patients showing post-intervention 7-point improvement in the PCI scores between the intervention group and the control group using Fisher’s exact tests. A linear mixed-effect model for repeated-measures analysis will be used to analyze the changes in cognition such as treatment, time point, and the interaction between treatment and time point. The between-group effect size will be calculated in post-treatment means (dividing the between-group difference with the pooled standard deviation). The effect sizes of 0.20, 0.50, 0.80, and 1.30 represent small, medium, large, and very large effect, respectively [41]. Two-sided significance level for all tests is 0.05.

Linear regression analysis adjusted for baseline will be used to assess the association between changes in cancer-related symptoms (fatigue, anxiety, and depression) and changes in cognitive function. Adherence to the Baduanjin exercise will be noted by adherence rate ((total frequency − absenteeism)/total frequency × 100%).

### Dissemination

The findings of this study will be reported in a master’s thesis by the main author and submitted to a peer-reviewed journal for publication. It will be presented at relevant conferences on the subject of matter if possible.

## Discussion

Currently, although cognitive decline is a well-known phenomenon, there are no pharmacological interventions to prevent or alleviate cognitive problems in patients with various non-central nervous system cancers. Physical exercise has been recommended as a part of rehabilitation, which appears to be a feasible and promising non-drug intervention that improves cognitive abilities. Preclinical study [42] has shown that low-intensity exercise may assist in preventing cognitive dysfunction in chemotherapy-exposed patients with breast cancer. Baduanjin exercise as a mind-body therapy has beneficial effects on fatigue, quality of life, and negative mood among patients [30, 43, 44]. In addition, this kind of exercise is easy to learn and can be widely used in the community with no limitation for the environment [45]. Meanwhile, there is evidence demonstrating improved attention of the elderly or patients with MC [46, 47]. In light of the previous literatures, we hypothesized that the exercise program may have a protective effect on the cognitive function of breast cancer survivors. Given ethical reasons, patients assigned into the control arm have the opportunity to carry out a 3-month exercise program after completion of the study, so as to decrease subsequently the risk of sample contamination. If Baduanjin exercise seems to be effective, it may provide evidence-based intervention during cancer treatment to possibly minimize or decrease cognitive problems, thereby improving patients’ quality of life. Previous preclinical and human studies have shown that a wider range of chemotherapy was associated with cognitive changes in cancer patients, so we hope that the results of this study will also be applicable to patients with other types of tumors during receiving chemotherapy. Therefore, the ultimate purpose of this study may have a positive impact on the quality of life or physical and mental health of the growing survivors with cancer.

## Trial status

Recruitment of patients started on 1 June 2020. The trial is currently underway and expected to be completed by 1 June 2021. The protocol is 1.0 version. The date of edition was 22 May 2020.

## Supplementary Information

**Additional file 1:.** SPIRIT 2013 checklist: recommended items to address in a clinical trial protocol and related documents.

## Data Availability

Data sharing is not applicable to this trial as no database is generated or analyzed for the current study.

## Abbreviations

FACT-Cog: Functional Assessment of Cancer Therapy-Cognitive Function
PCI: Perceived cognitive impairment
PCA: Perceived cognitive ability
HVLT-R: Hopkins Verbal Learning Test-Revised
TMT: Trail-Making Test
DSST: Digit Symbol Substitution Test
MFSI-SF-C: Chinese version of Multidimensional Fatigue Symptom Inventory-Short Form
HADS: Hospital Anxiety and Depression Scale
FACT-B: Functional Assessment of Cancer Therapy-Breast
SAE: Serious adverse event
TOC: Trial Oversight Committee
